# Assessing cognitive components of computational thinking

**DOI:** 10.3389/fpsyg.2025.1434453

**Published:** 2025-09-10

**Authors:** Andrew J. Mertens, Eliana Colunga

**Affiliations:** Department of Psychology and Neuroscience, University of Colorado, Boulder, CO, United States

**Keywords:** computational thinking, Computer Science, computer science education, cognition, programming, decomposition, sequencing, abstraction

## Abstract

Recent years have seen a dramatic increase in Computer Science (CS) education programs implemented at the K-12 level. This emphasis on CS education comes not only from the fact that computer skills are becoming an ever-more integral part of modern life, but also from a notion that learning how to program facilitates the development of a set of more general skills and strategies collectively known as Computational Thinking (CT). What makes CT special is the fact that it can be applied in an array of different contexts that are not limited to the CS domain. The present work adapts tasks from established cognitive tests in an attempt to capture some of the components specifically theorized to comprise CT, namely decomposition, sequencing, and abstraction. To test this, we conducted three studies to test the degree to which these measures relate to proficiency and experience with computer programming. Study 1 examines this relationship in 8–12 year-old children enrolled in STEM summer camps. Study 2 examines the programming proficiency-CT relationship in a different population and setting: fourth graders in a public elementary school. Study 3 aims to contribute converging evidence for the relationship by measuring CT and programming experience in an online study in the 8–12 year-old STEM summer camp population. The results reveal that performance on the decomposition measure consistently correlates with both proficiency and experience measures of programming in young children. We discuss these findings in the context of a potential progression for the emergence of CT-related skills throughout development.

## 1 Introduction

Computational thinking (CT) is a method of reasoning and problem solving that allows one to effectively interface with computers (Wing, 2014). The general nature of the skills that CT encompasses has led some in the Computer Science Education (CSEd) field to speculate that they may be beneficial in contexts extending beyond computer competency (Wing, 2006; Resnick et al., 2009; Barr and Stephenson, 2011). This notion that CSEd skills can have a wide influence outside of Computer Science is supported by findings that computer programming training results in varying degrees of improvement in domains such as mathematics, reasoning, and general academic achievement (Scherer et al., 2019). However, the breadth of the measures used in these studies is often incongruous with the specific components of CT that are discussed in the CSEd literature. That is, there seems to be little collaboration between those who measure transfer effects that might be attributed to CT and those who are concerned with defining and measuring CT. In the present work, we identify cognitive tests as potential candidates for measuring three specific CT components identified in the CSEd literature: decomposition, sequencing, and abstraction. The measures we identify were not designed to measure CT components so their validity as measures of these components is not known. We then evaluate how strongly these components relate to programming proficiency in two learning settings: a children's STEM summer camp program and an extra-curricular program in a fourth-grade classroom.

## 2 Background

Although the term “Computational Thinking” was originally coined in Seymour Papert's book, *Mindstorms; Children, Computers and Powerful Ideas* (Papert, 1980), it was more recently popularized and, to a degree, reconceptualized in Wing (2006). In the present paper, the term CT will be used to refer to Wing's reconceptualized view of the construct, as it is most readily recognized today in CSEd. Distinct from programming, the Wing (2006) conceptualization of CT does not merely represent an individual's ability to create computer programs, but rather a more fundamental way of thinking that arises from and, in turn, can facilitate the development of skills like programming.

Wing (2006) describes three characteristics that will help guide the present discussion of CT. First, CT consists of the fundamental skills that allow for the creation of programs, not merely the ability to write code. That is, CT is domain-independent. Second, using computers as a programmer results in the development of CT. Finally, everyone has, uses, and can further develop CT to some degree, regardless of programming experience, again highlighting the domain-independent nature of CT. Within the CSEd community, this description prompted suggestions that such fundamental skills learned through computer programming could transfer to a broad array of contexts and disciplines (Resnick et al., 2009; Barr and Stephenson, 2011).

In trying to understand and characterize Computational Thinking, CSEd scholars and organizations such as Google and the International Society for Technology in Education (ISTE) have proposed different frameworks and a number of specific CT components. For example, Brennan and Resnick (2012) provide a framework of CT that includes three tiers of aspects of CT: concepts (e.g., sequences, loops, parallelism, events, conditionals,), practices (e.g., debugging, reusing, abstracting, and modularizing), and perspectives (e.g., expressing, connecting, and questioning). The International Society for Technology in Education (ISTE) and the Computer Science Teachers Association (CSTA) made a collaborative effort in 2011 to arrive at a description of CT in the K-12 setting. A committee of higher education researchers, K-12 administrators, and teachers identified skills and traits as components of CT in the K-12 setting. These are skills such as formulating problems in a way that enables us to use a computer to solve them, representing data through abstractions such as models and simulations, automating solutions through a series of ordered steps, and generalizing and transferring the problem solving process, (for full summary see ISTE and CSTA, 2011). In the present work we select three fundamental components of computational thinking that have both clear cognitive underpinnings and wide representation within the proposed frameworks mentioned above and we propose domain-independent measures for each of them.

Guided by these extensive descriptions, a number of studies have been designed around the measurement of various CT components. Many of these studies infer CT through proficiency in tasks directly related to Computer Science (CS); for example, evaluating the diversity of functions in a child's code or asking children to reflect on their thinking process as they wrote code in an interview (Brennan and Resnick, 2012). That is, CT skills are most often measured in the products of programming (i.e., code) or through self-report about the programming experience itself, (see Lye and Koh, 2014, for a review). It is also common practice to assess CT by evaluating performance on tasks or problems that closely resemble coding. For example, Grover et al. (2015) designed a course that included programming assignments in Scratch, a block-based programming language designed for children, and assessed CT afterwards using test questions about snippets of Scratch code. The majority, though not all, of the items on the Computational Thinking Test described in Román-González et al. (2017) also include multiple choice options containing snippets of Scratch-like code blocks. Simply put, CT often is not measured in terms of the more fundamental, domain-independent skills that (Wing, 2006) originally claimed make up the core construct that is CT. It is precisely the development of such skills, however, that may underlie the transfer effects proposed in the CSEd literature.

Even before (Wing, 2006) popularized the term *Computational Thinking*, the idea that learning computer programming could result in benefits to domains outside of CS had been examined extensively. More specifically, a whole body of work measuring improvement in a variety of cognitive and academic skills through programming experience predates the recent resurgence of CT by over a decade (for a review, see Liao and Bright, 1991). Scherer et al. (2019) conducted a meta-analysis of this work, encompassing 105 published papers and theses (over 500 effect sizes) dating from 1973 to 2017. This work looked at the effects of learning different programming languages (e.g., logo, basic, scratch, etc) on a variety of standardized and non standardized measures of creativity and intelligence, as well as mathematics, science, and language. The Scherer et al. (2019) meta-analysis provides evidence that computer programming experience can, in fact, result in both the improvement of a number of basic cognitive skills (e.g., fluid intelligence and spatial skills) as well as other more specific domains such as mathematics, with effect sizes ranging from –2.02 to 8.63. Notably, the meta-analysis also found that experience in domains such as literacy showed no effect of improving cognitive skills.

As Scherer et al. (2019) note, although distinct from CT, the programming skills included in their analysis overlap considerably with CT. That is, CT can be conceptualized as a set of skills responsible for the observed benefits of learning how to program. Based on this overlap, the transfer of programming proficiency reported by Scherer et al. (2019) could be taken as partial support for claims of CT transfer posited by the CSed literature. However, it seems that these transfer studies do not make much contact with the CSed literature, as evidenced by the fact that few of the studies reviewed in Scherer et al. (2019) make any mention of the term “computational thinking.” However, there are some examples in which the relationship between cognitive abilities and CT is assessed within the CSed literature. Román-González et al. (2017), for example, include an analysis of the relationship between their Computational Thinking Test and three broad cognitive abilities: fluid reasoning, visual processing, and short-term memory. In other studies, cognitive assessment measures are used as direct measures of CT components. For example, Rowe et al. (2021) uses Raven's Progressive matrices as an index of abstraction alongside non-clinical puzzle formats to measure decomposition, pattern recognition, and algorithm design.

One difficulty shared by all studies investigating the cognitive aspects of CT, including this one, is that, generally, cognitive constructs (e.g., fluid reasoning) are too broad to cleanly map onto the relatively specific CT components described in the CT literature (e.g., decomposition). Many of the studies surveyed in Scherer et al. (2019), for example, measure transfer using tasks encompassing similarly broad cognitive skills, (e.g., problem-solving; Dalton and Goodrum, 1991; Degelman et al., 1986). The same can be said of some of much of the work cited above that attempts to assess the cognition-CT relationship, (i.e., Román-González et al., 2017; Rowe et al., 2021). Because of this, it is difficult to infer which CT components suggested by the CSEd literature are involved in the transfer effect and to what extent. In the present studies, we aimed to adapt cognitive measures that selectively relate to specific CT constructs. However, our measures are also susceptible to this specificity issue to some degree (see Section 13 for further discussion).

There are a few studies that do try to bridge this divide in the literature by examining programming ability in association with cognitive components that, to some degree, resemble those identified in the CT literature. Swan et al. (1991), for example, measures improvement in, among other constructs, subgoal formation and trial and error, which might be considered akin to the CT skills of decomposition and iterative design. Swan et al. (1991) found that 4–6 grade students who received problem solving instruction in LOGO programming showed more improvement in these skills than children in a control condition that practiced the same problem solving strategies in an activity involving paper cutouts. An example that adheres more closely to using CT constructs is the work of Kazakoff et al. (2012) on the development of Kindergarten and pre-K children's ability to order a set of pictures to form a narrative (i.e., sequencing). In their experiment, children were tested before and after participating in a 1-week robotics curriculum, revealing a significant improvement in sequencing skills. A group of children receiving no robotics training showed no significant improvement (Kazakoff et al., 2012). These works require replication and deserve to be extended upon in a way that incorporates the modern conceptualization of CT and expands the number of cognitive components examined. The present work studies CT development in elementary school age children using measures representing multiple CSEd-recognized CT components with well-established cognitive construct counterparts: decomposition, sequencing, and abstraction.

## 3 Current studies

Given the literature reviewed above, we know that CS experience should result in the improvement of certain abilities. The cognitive components chosen to represent those abilities in this work were selected based on their prevalence in the CSEd literature on computational thinking. Once these components were identified, we selected existing cognitive tasks developed for clinical assessment and adapted them to measure these CT components and test their relationship with programming proficiency in elementary school children. The three computational thinking components we selected were decomposition, sequencing, and abstraction. We conducted two studies to test the degree to which these cognitive skills often associated with CT relate to CS experience as measured through programming proficiency. We then conduct one additional study to test the degree to which these cognitive skills relate to a different but related measure of CS experience, namely the number of different ways participants have been exposed to programming.

*Decomposition* refers to an individual's capacity to deconstruct a complex problem or system into parts that make it easier to solve or understand. Decomposition is commonly cited as an integral part of CT. It is listed by name in the original Wing (2006) conceptualization and in Barr and Stephenson (2011), and indirectly referred to as “modularization” in Brennan and Resnick (2012). The concept of decomposition is also alluded to in a definition of CT developed by the International Society for Technology in Education (ISTE) and the Computer Science Teachers Association (CSTA), which includes “formulating problems in a way that enables us to use a computer or other tools to solve them” (ISTE and CSTA, 2011). In our decomposition task, children are given a series of geometric pictures and asked to reproduce them by putting together their component parts.

*Sequencing* is another skill referenced by Brennan and Resnick (2012) in direct association to CT. Sequencing is crucial for automation and algorithmic thinking, which are included in the Barr and Stephenson (2011) description of CT and in the CT definitions from ISTE and CSTA (2011). Sequencing, as it is used in the present paper, is conceptualized as the skill required to make inferences about cause and effect to logically order tasks or events. In our sequencing tasks, children are given picture cards and asked to put them in the right order.

Finally, *abstraction* refers to one's ability to learn a general concept from specific examples or instances and apply that concept in distinct contexts. Abstraction is identified by Brennan and Resnick (2012) as well as Wing (2006) as a critical aspect of CT. Wing (2017) states that “the most important and high-level thought process in computational thinking is the abstraction process.” Abstraction is also referred to in the ISTE and CSTA definition which includes “generalizing and transferring (the CT) problem solving process to a wide variety of problems” (ISTE and CSTA, 2011). In our abstraction task, children are asked to find the rule implicit in a pattern of geometric pictures.

All three studies examine these constructs—decomposition, abstraction, and sequencing—and their relationship to programming proficiency. Study 1 employs a pre-post design to measure the CT components reviewed above, before and after participating in week-long summer camps focusing on topics in either CS or other STEM fields. The pre-post nature of the design allows the investigation of a secondary research question, namely a direct examination of the claim that CT skills undergo more change through CS experience than other types of experience. If learning how to program improves CT, then we might expect children to show more improvement in CT measures after a week of CS instruction compared to other similarly enriching STEM activities.

Study 2 uses a single-visit design to replicate the relationship between programming proficiency and performance on the CT tasks in a different population: a class of fourth-grade students. Unlike the participants in Study 1, the children in this study are not self-selected by their interests in STEM camps and have a relatively uniform level of educational experience which includes little to no formal CSEd instruction.

Study 3 is conducted online in a single session using a separate programming measure of programming experience rather than programming proficiency. It aims to contribute converging evidence for the relationship between our CT measures and programming in a sample of 8-12 year-old STEM summer camp attendees drawn from the same population as study 1.

## 4 Study 1 method

### 4.1 Participants

Fifty-three children (26 males, 27 females) between the ages of 5 and 12 (*M* = 9.23, *SD* = 1.29) years were recruited from 14 different week-long, STEM-focused summer camp programs. Parental consent and child assent to participate were both obtained in writing at the time of data collection. These camps were focused on a number of topics, some directly related to CS (e.g., robotics, video game design, coding) and others related to other STEM fields (e.g., chemistry, engineering, physics). Thirteen children were recruited from CS-related camps and 33 were recruited from non-CS camps (see Table 1 for more detailed summary). Participants were given $10 USD and a children's book as compensation for their time.

**Table 1:** A breakdown detailing the number of participants in each specific camp, age range, and camp type that were included in analysis.

**Camp type**	**Age range**	**Camp name (*n*)**
Control camps *n* = 33, 20 male	Grades 2–4	If you build it (3)
		(Not so) mad scientists (6)
	Grades 3–5	Muggle magic: science of Harry Potter (4)
		Paint and pixels: innovative iPad art (4)
		Phun with physics (3)
	Grades 4–6	Animation (3)
		Amazing race (2)
		Board game design (3)
		Escape room (5)
Coding camps *n* = 13, 6 male	Grades 2–4	Robot playground (4)
	Grades 3–5	Kid coders: scratch stories and games (2)
	Grades 4–6	Minecraft adventure (4)
		Bloxels video game design (3)

### 4.2 Measures

**Figure 1:**
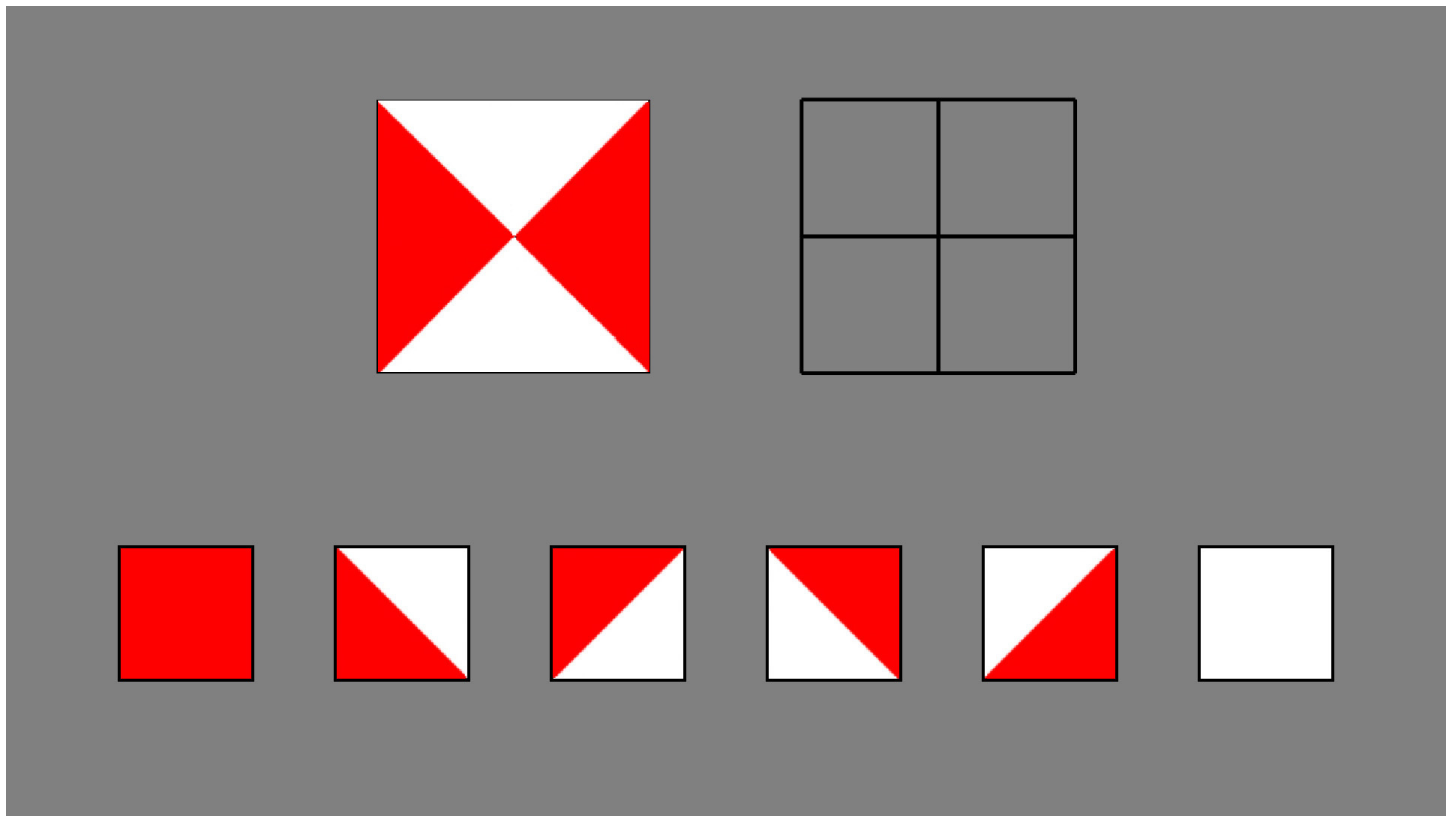
An screenshot of a trial from the computer adaptation of the Block Design task. In these computer adaptations, participants arrange virtual cards by clicking an image in the bottom row to select it and then clicking the black boxes above to move their selected image to that location. Stimulus images adapted from Wechsler et al. (1992), with permission from Pearson PLC.

Computer versions of subtests from established cognitive batteries, the WISC-III (Wechsler et al., 1992) and Leiter-R (Roid and Miller, 2011), were used to evaluate the three components of CT identified above (see Table 2 for a summary). We adapted our decomposition measure from the “Block Design” task of the WISC-III, which was designed to measure general visual-spatial ability. The task requires one to recreate a pattern piece by piece by arranging a set of squares with different color patterns (see Figure 1). The need to break up the target pattern in order to recreate it makes this task an appropriate measure of decomposition.

**Table 2:** A table describing the cognitive measures used, their original sources, and the CT constructs they represent.

**Construct**	**Subtest name**	**Description**	**Cognitive battery**	**Number of items**
Decomposition	Block design	A 2-D pattern must be replicated using a set of colored tiles	WISC-III	1 practice, 8 test
Sequencing	Picture arrangement (Study 1)	A set of images, in a mixed-up order, must be rearranged to create a plausible story	WISC-III	1 practice, 14 test
	Sequential order (Study 2)	A logically progressing sequence of symbols must be completed using a selection of symbols that includes distractors	Leiter-R	1 practice, 10 test
Abstraction	Repeated patterns	A partial pattern must be completed using a selection of symbols among distractors	Leiter-R	1 practice, 6 test

**Figure 2:**
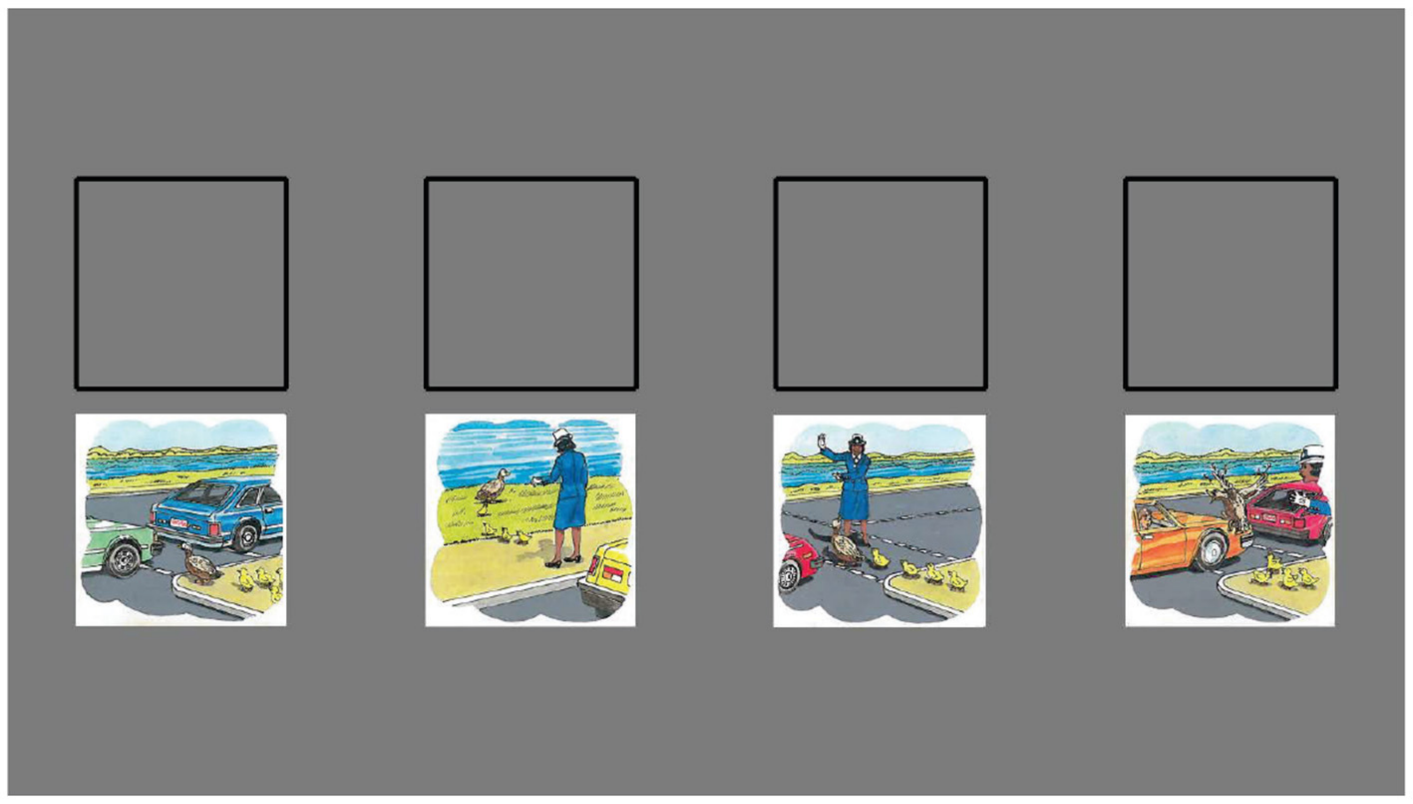
A screenshot of a trial from the computer adaptation of the Picture Arrangement task. Stimulus images adapted from Wechsler et al. (1992), with permission from Pearson PLC.

The “Picture Arrangement” task, also adapted from the WISC-III, was used to measure sequencing. In this task, participants are asked to place scenes depicted on cards in chronological order (see Figure 2). Being designed to measure reasoning ability and requiring one to use an understanding of cause and effect, we consider it to be a reasonable measure of sequencing.

**Figure 3:**
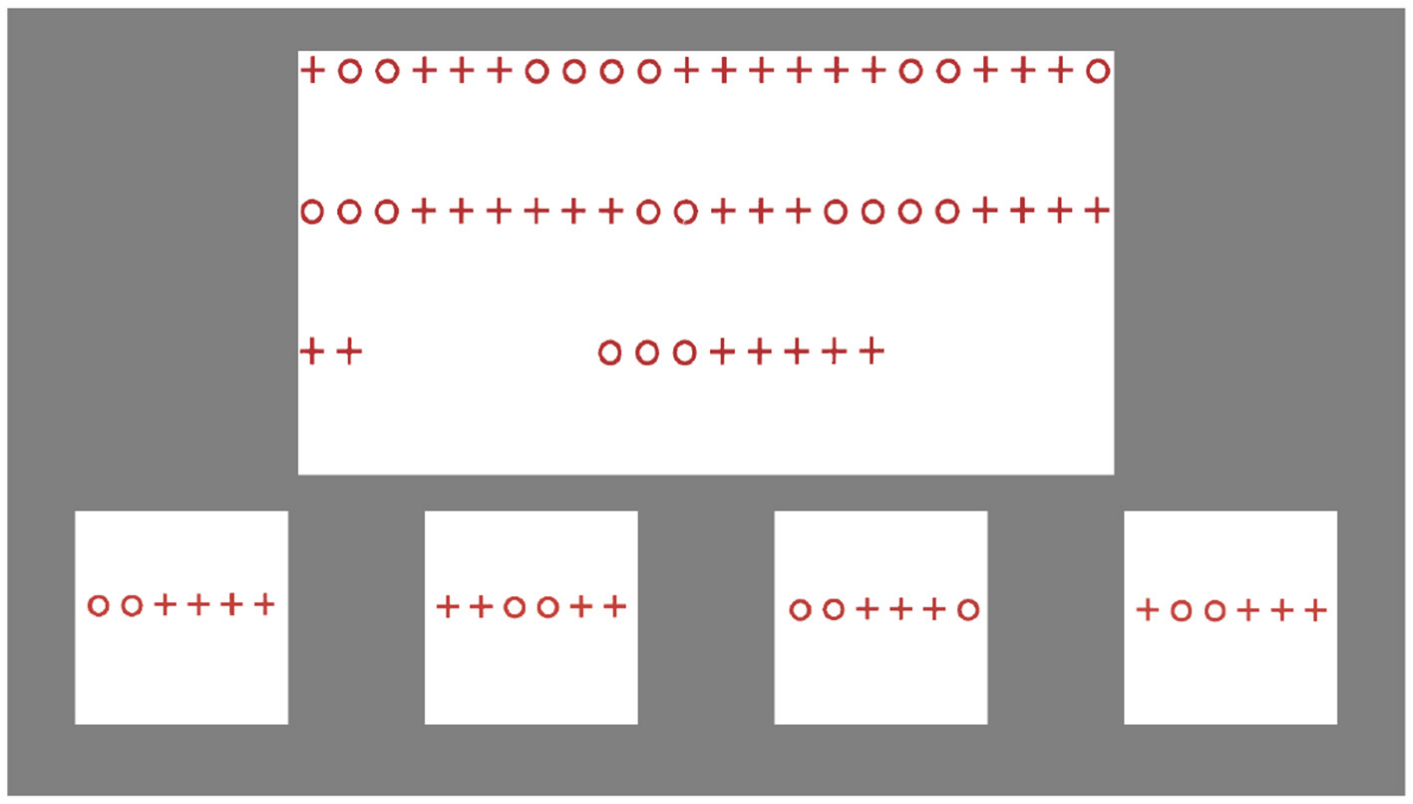
A screenshot of a trial from the computer adaptation of the Repeated Patterns task. Stimulus images adapted from Roid and Miller (2011), with permission from Stoelting.

The “Repeated Patterns” task from the Leiter-R, one of two tasks included in the battery's fluid reasoning composite score, was adapted as our measure of abstraction ability. In this task, participants are shown a sequence of objects or shapes that establishes a pattern and are asked to continue the pattern using an assortment of cards, some which fit the pattern and some that do not. Some trials require the participant to apply rules gleaned from existing parts of the pattern to missing parts of the pattern that represent previously unobserved contexts. For example, one trial requires the participant to recognize a pattern that consists of sequences of different numbers of “+” and “o” symbols (i.e., 2 o's followed by 3 +'s, followed by 4 o's, followed by 6 +'s, followed by 2 o's). However, this sequence wraps from one line to the next on the test card, so the participant has to know to apply this pattern even when groupings of symbols are divided in ways they have never seen before (i.e., if a line ends with 4 +'s and the next begins with 2 +'s, the participant much recognize that the next part of the sequence must start with 2 o's). Figure 3 depicts a screenshot of this trial. This aspect of the task that involves abstracting pattern rules to unfamiliar contexts makes it a reasonable measure of abstraction.

Items from these tasks were adapted for computer administration to facilitate the simultaneous testing of multiple subjects within the relatively short period of time allotted before and after the camps. Participants would arrange the virtual cards in these computer adapted tasks by first clicking on a card to select it and then clicking one of multiple outlined spaces on the screen designating where cards can be placed to complete the task. These computer versions of the tasks were administered using PsychoPy behavioral testing software (Peirce et al., 2019).

Each of the measures described above consists of a series of items of increasing difficulty. As such, to create separate versions for this pre-post design, the items of each of the CT measures were divided into two equal sets in alternating fashion (e.g., items 1, 3, 5... in one set and items 2, 4, 6... in the other). The order in which the two sets were administered was counterbalanced across the two testing sessions. Accuracy and response time data were collected for each item of each CT measure. Scores of individual items from the WISC-III subtasks were calculated by assigning a number of points to correct responses depending on the response time, as directed in the WISC-III manual (Wechsler et al., 1992). Item scores were then summed to create a single performance score for each version of the measure. Item scores from the “Repeated Patterns” measure were calculated by summing the number of pattern segments that were correctly placed across all trials.

In addition to performance in our cognitive tasks, we measure performance in Lightbot, a puzzle videogame designed to teach programming concepts.^1^ This measure was chosen partially for the low floor of the game's learning curve, allowing even children with no programming experience to progress through the first few levels. In it, players are tasked with navigating their avatar to specific locations through terrain that becomes increasingly complex to traverse as the game progresses through a series of levels. This is achieved by arranging sequences of icons representing basic actions for the avatar to perform such as moving, turning, and jumping. Once a player has input a series of these commands, they can run the sequence and see the avatar act them out. If the avatar fails to complete the objective given the sequence dictated by the player, the sequence stays on the screen and the player is free to adjust the commands before trying again. The game was designed to teach programming practices including planning, testing, and debugging as well as programming concepts such as sequencing, procedures, and loops (Yaroslavski, 2014). In their evaluation of Lightbot, Gouws et al. (2013) reports that the game succeeds in representing a number of programming concepts, particularly processes, transformations, models, abstraction, and, to a somewhat lesser degree, patterns and algorithms. Screen recordings of participants' gameplay were reviewed to measure their total progression by the end of the session. Progression was defined as the number of consecutive levels participants completed within the first 4.8 minutes, the minimum amount of time that any participant was allowed to play the game.

We include two additional measures of general cognition that are not theorized to be directly related to CT in order to establish the selectivity with which our measures capture CT skills. One of these is the Test for Audible Comprehension of Language (TACL)-Fourth Edition (Carrow-Woolfolk and Allen, 2014), a measure of English comprehension. In each item of the TACL, three pictures are presented and a sentence is read aloud by an experimenter. Participants are asked to non-verbally indicate which of the three pictures the sentence describes. In the present study this was done in groups, with participants viewing the pictures on a projector screen and marking the appropriate picture on individual paper copies of the pictures while an experimenter read the sentences. Accuracy was calculated by summing the number of correct responses. The other measure we included is a version of the Track-It task (Fisher et al., 2013), which measures sustained attention. At the start of each trial of the Track-It task, a target abstract shape is marked as the target among six different abstract distractor shapes. Each shape is placed on a random space within a grid, which remains visible for the duration of the trial. Once the subject presses the spacebar to indicate they are ready, all of the shapes move around on the grid for a minimum of five seconds before disappearing. Subjects are then asked to specify the last grid space in which the shape was seen before disappearing.

### 4.3 Procedure

Data collection occurred in two sessions totaling about one and a half hours in duration. The first session was completed during the 30 minutes immediately following check-in for the first day of camp. During this session, participants were randomly assigned to complete one of two versions of the CT measures individually on computers using mouse and keyboard. At both time points, participants were informed that they could skip any given trial of a CT task by clicking on the button to go to the next trial. Participants completed the second session immediately following the final day of attending 5 days of STEM summer camp for 7 hours per day. Participants individually completed the versions of the CT tasks that they had not encountered in session one. Participants were then asked to play the Lightbot game on Apple iPads for the remaining duration of the session to measure programming proficiency. Screen recordings of the Lightbot gameplay were saved.

## 5 Study 1 results

### 5.1 CT in STEM summer camps

Due to the strict time constraints imposed by the schedules of the camps and a delay of testing due to some late arrivals, not all participants were able to complete each measure of the experiment. For this reason, children were excluded from some analyses and not others on the basis of their completion of the measures included in those analyses. Of the 53 participants recruited, seven were excluded from analysis entirely: four completed only the first visit, two completed the same measure version during both visits due to experimenter error, and one (age 5.66 years) was outside our target age range of 7–12 years. Time limitations prevented an additional 17 participants from reaching the programming proficiency measure at the end of the second session, leaving only 29 participants between 7 and 12 years of age (*M* = 9.58, *SD* = 1.23) to be included in the analysis described in Section 5.2. The 46 participants between 7 and 12 years of age (*M* = 9.37, *SD* = 1.2) who completed at least one CT measure in both visits and were not excluded from analysis for the reasons listed above are included in the following analyses.

First, we fit an omnibus mixed effects regression model to the data to examine whether CT scores differed between the start of the camp week and the end of the camp week. Because the scoring methods used in the WISC-III and Leiter-R differ, CT scores were standardized within their respective subtask before being combined into a single variable representing CT scores across the three measures. This combined variable was regressed on fixed effects of age, version order (A–B/B–A), and the main effects and interaction terms between CT measure (decomposition/sequencing/abstraction), time-point (pre/post), and condition (coding-related camp/non-coding camp) while treating participants as a random effect (modeled using the R lmerTest package; Kuznetsova et al., 2017). An analysis of variance of this model using Satterthwaite's method is shown in Table 3. Including the sustained attention measure as a covariate yielded the same pattern of results.

**Table 3:** Analysis of variance using Satterthwaite's method to examine influence on CT scores during STEM summer camp attendance.

	**Type III sum of squares**	**df Num**.	**df Denom**.	***F***	***p***
**Main effects**					
Age	14.96	1	42	24.19	< 0.001
Test version	0.01	1	42	0.01	0.905
CT measure	0.13	2	217	0.11	0.897
Time point	0.97	1	214	1.56	0.212
Condition	0.50	1	42	0.80	0.375
**Interactions**					
CT measure × time point	0.51	2	214	0.41	0.661
CT measure × condition	0.29	2	217	0.23	0.791
Time point × condition	1.80	1	214	2.91	0.090
CT measure × time point × condition	0.44	2	214	0.35	0.702

**Figure 4:**
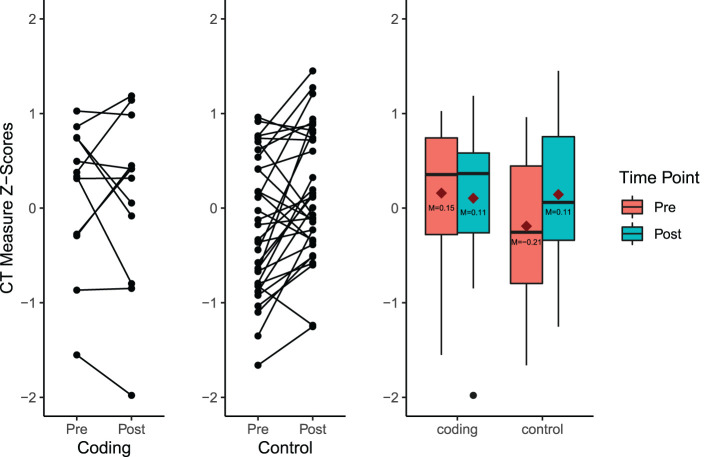
Plots demonstrating the interaction between condition and time point, characterized by an improvement in CT performance among attendees of non-coding camps. Red diamonds represent group means.

The interaction between time-point and condition was of particular interest in this analysis, which would indicate whether participants in coding-related camps exhibited a greater change in CT performance than those in non-coding camps. However, the results reveal that this interaction reaches only marginal significance, *F*(1, 214) = 2.91, *p* = 0.090. Mean CT measure *z*-scores in the non-coding camp condition improve from visit one (*M* = –0.21, *SD* = 0.96) to visit two (*M* = 0.11, *SD* = 1.00) while scores in the coding camp condition decrease from visit one (*M* = 0.15, *SD* = 0.93) to visit two (*M* = 0.11, *SD* = 1.07; see Figure 4).

### 5.2 Relation between CT and programming proficiency

**Figure 5:**
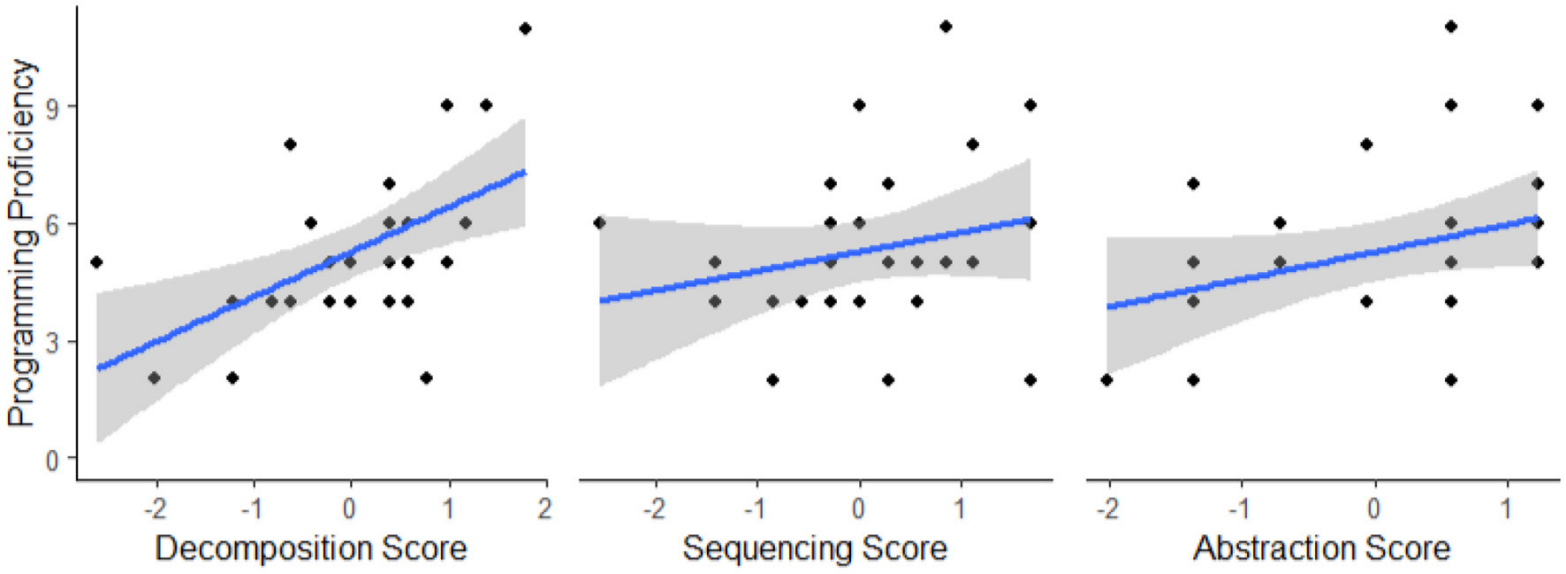
Scatterplots showing relationships between programming proficiency and standardized CT measure scores in study 1. Gray shading represents the 95% confidence interval.

Next, we investigate the relationship between measures of cognitive CT constructs and programming proficiency by fitting a multiple regression model. To this end, we conduct a multiple regression analysis fitting programming experience to predictors representing main effects for each of the CT measures as well as a predictor for the main effect of age. The scores of each CT measure were standardized using *z*-score transformations and age was mean-centered. While holding fixed the main effects of sequencing, abstraction, and age, the model reveals a significant main effect of decomposition β = 1.23, *t*(24) = 2.14, *p* = 0.010 (see Figure 5). No significant main effect of sequencing score (*p* = 0.910), abstraction score (*p* = 0.194), or age (*p* = 0.184) were revealed. Correlations between CT measures revealed no relationship between decomposition scores and abstraction scores *r*(27) = 0.318, *p* = 0.092 or between sequencing and abstraction scores *r*(27) = –0.03, *p* = 0.867. A moderate positive relationship was revealed between decomposition and abstraction scores *r*(27) = 0.47, *p* = 0.011.

Not every participant who completed the CT measures was able to complete the non-CT cognitive tasks TACL and Track-It due to time constraints. For this reason, main effect terms for TACL and Track-it were excluded from this initial model. We conduct an additional analysis using a model that includes these measures and find that the main effect of decomposition score is maintained with the variance explained by TACL and Track-It removed β = 1.58, *t*(21) = 3.61, *p* = 0.002. Neither TACL nor Track-it, our two general cognitive control conditions, are positively correlated with programming proficiency. In fact, we find a negative effect of Track-It score β = –0.84, *t*(21) = –2.25, *p* = 0.035 and no effect of TACL score (*p* = 0.595).

## 6 Study 1 discussion

In our investigation of changes in CT scores from visit one to visit two, we observe that CT scores in the coding camp condition stay about the same and CT scores in the non-coding camp increase. Although this result did not match our expectations, given that CT is not exclusive to coding experience and that many other forms of activity are likely to draw on and improve CT skills, it is not entirely surprising that CT skills among non-coding camp attendees improves significantly over the course of one week of summer camp sessions.

This set of findings indicates that, of those examined here, decomposition is the only CT component that correlates with programming proficiency across the various levels of prior CS experience represented in the sample. The general lack of improvement in CT ability as a result of attendance in STEM summer camps is a possible indication that the time course of CT development is relatively slow, resulting in a lack of observable change in the relatively short instruction period of one week. A second study is described in Section 7 which aims to extend these findings by examining the same relationship between our cognitive CT components and programming ability in a sample of public elementary school students.

## 7 Study 2 method

### 7.1 Participants

Thirty-one children (14 males, 17 females) between the ages of 9 and 11 (*M* = 9.83, *SD* = 0.36) were reruited from a class of fouth grade students at a public elementary school in the Boulder Valley School District. Prior to participation, parental consent to participate was obtained via permission form. Child assent to participate was also obtained in writing at the time of data collection. In this school, 17.70% of students are in an English Language Learner (ELL) program and 25.00% are eligible for free or reduced-cost lunches. As compensation for participating in this study, we offered the class a collection of children's books for the classroom.

### 7.2 Measures

**Figure 6:**
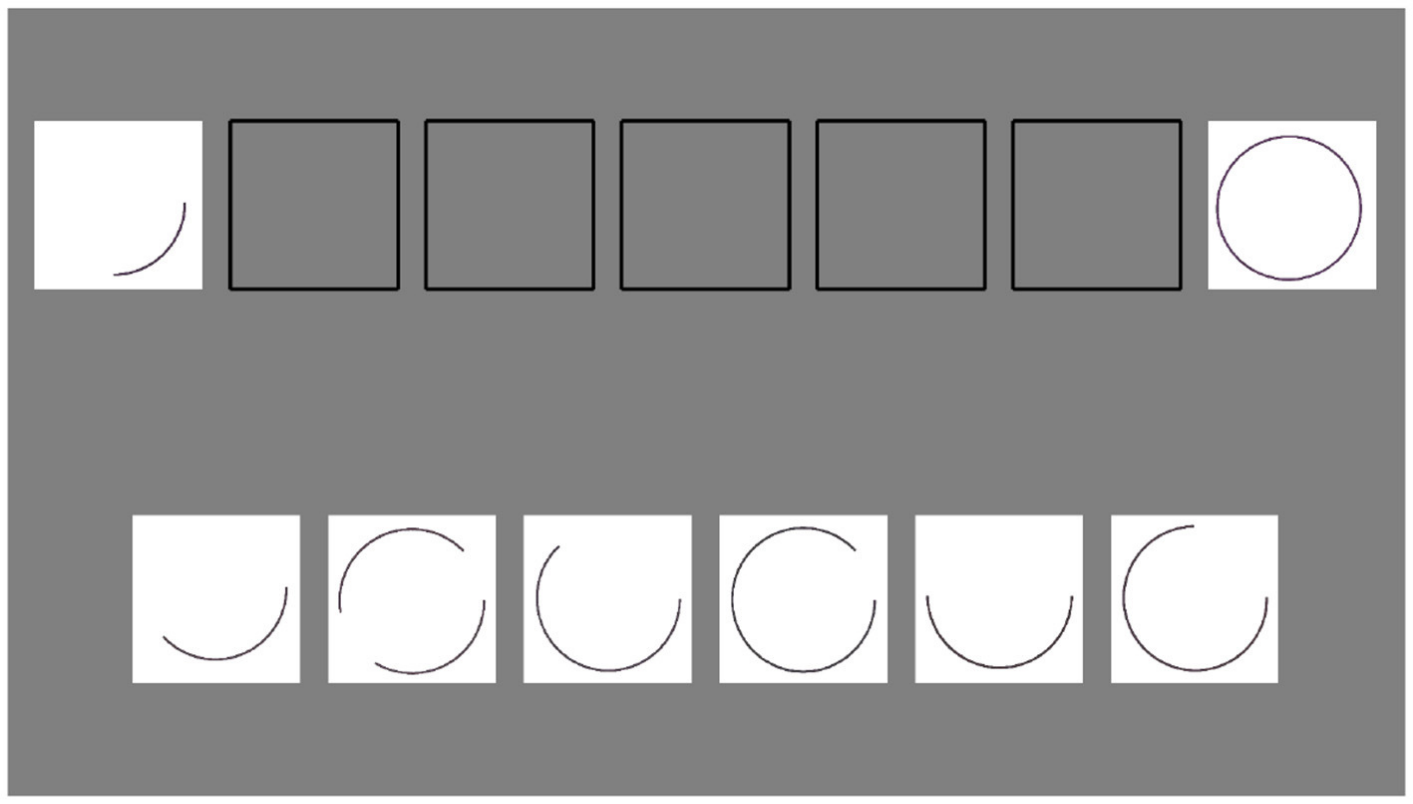
An screenshot of a trial from the computer adaptation of the Sequential Order task. Stimulus images adapted from Roid and Miller (2011), with permission from Stoelting.

The tasks used to measure decomposition and abstraction in Study 1 (“Block Design” and “Repeated Patterns,” respectively) were used once again in Study 2. In lieu of the WISC-III Picture Arrangement task used to measure sequencing in Study 1, this study employs a computer adaptation of the “Sequential Order” subtask from the Leiter-R battery, which is the other measure included in the fluid reasoning composite of the Leiter-R along with “Repeated Patterns” (Roid and Miller, 2011). Like the “Picture Arrangement” measure, the “Sequential Order” task requires the participant to put a series of images into a logical order. The main advantage of the “Sequential Order” task is that the images seen on the cards are abstract designs rather than real-world scenes, allowing one to complete the task with less prior world knowledge than the WISC-III's “Picture Arrangement” task requires (see Figure 6). The act of arranging sequences of these abstract designs may appear quite different from arranging depictions of real-life scenes (as in the Picture Arrangement task) on a surface level, but at their core, these tasks capture the common skill of sequencing, namely ordering items based on logical inference. Whether the underlying logic relies on understanding of real-world cause and effect or understanding an underlying rule governing a sequence, the ability to order items based on that logic is the common ability that we intend to measure. Although we believe the WISC-III's “Picture Arrangement” task successfully captures this type of logical inference, success in that task also depends heavily on an understanding of the intentions of the agents involved and, in some cases, cultural conventions and references, which might have been less readily accessible to the participants of this study.

The three CT measures used in this study consist of the full subtask, rather than being split into two versions as in Study 1. Scoring for the remaining subtask from the WISC-III, “Block Design,” was conducted as described in Section 4.2 of Study 1. With the measures consisting of the full subtask in this study, scaled scores for the Leiter-R subtasks were determined by the number of correct pattern segments and the participant's age, as directed in the Leiter-R manual (Roid and Miller, 2011).

The Lightbot programming game is administered once again, and the progression measure is extracted from screen recordings as a measure of programming proficiency. As in Study 1, progression was defined as the number of consecutive levels that participants completed within the minimum amount of time any one participant spent playing the game, which coincidentally matches the same cutoff used in Study 1: 4.8 minutes.

We include the Track-It task in study two in order to take into account the variance within the ELL population of our sample. We were forced to abandon the use of the Track-It task due to time constraints.

### 7.3 Procedure

Due to scheduling constraints from collecting data in a school setting, data for this study were collected in two sessions. In the first session, participants completed the three CT measures on Windows laptops using the keyboard and an external mouse. Participants first completed the new sequencing task, followed by the decomposition and abstraction tasks. In a separate session 1–2 weeks after the first, participants completed the TACL language comprehension task on pen and paper as an experimenter presented the items one-by-one on a projector screen and read the sentences aloud to the participants as a group. Finally, students were given the remainder of the second session to play the Lightbot programming game on Apple iPads.^2^

## 8 Study 2 results

**Figure 7:**
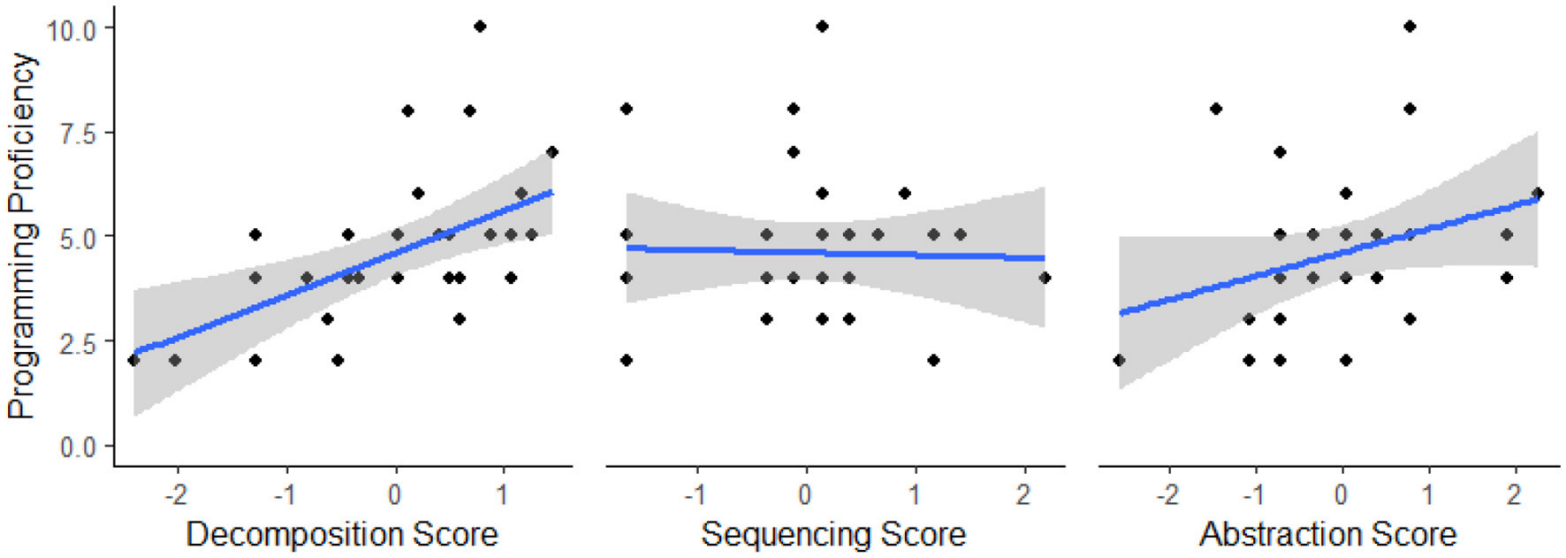
Scatterplots showing relationships between programming proficiency and standardized CT measure scores in study 2. Gray shading represents the 95% confidence interval.

As in Section 5.2 in Study 1, the analysis of data collected in Study 2 consists of an examination of the relationship between cognitive CT constructs and programming proficiency. Once again, we conduct a multiple regression analysis fitting programming experience to predictors representing main effects for each of the CT measures as well as a predictors for the main effects of language comprehension score and age. The scores of each CT measure and the language comprehension score were standardized using *z*-score transformations and age was mean-centered. While holding fixed the main effects of sequencing, abstraction, language comprehension, and age, the model reveals a significant main effect of decomposition β = 0.88, *t*(25) = 2.61, *p* = 0.015 (see Figure 7). No significant main effect of sequencing score (*p* = 0.074), abstraction score (*p* = 0.198), language comprehension (*p* = 0.378), or age (*p* = 0.451) were revealed. Correlations between CT measures revealed no significant relationship between decomposition scores and sequencing scores *r*(29) = 0.30, *p* = 0.096, between sequencing and abstraction scores *r*(29) = 0.26, *p* = 0.154, or between decomposition and abstraction scores *r*(29) = 0.24, *p* = 0.198.

## 9 Study 2 discussion

These results indicate that decomposition correlates with programming proficiency among fourth-graders with virtually no previous CS experience, suggesting a similar pattern of results to those observed in Study 1 but with a distinctly different population. Despite the use of a different measure, sequencing ability still appears not to be related to programming proficiency. The similar pattern of results between the two studies represents compelling support for the notion that decomposition is a prevalent part of CT development, at least at the ages examined here.

## 10 Study 3 method

### 10.1 Participants

Participants were 25 children between the ages of 8 and 12 (*M* = 9.60, *SD* = 0.78) years who were current or previous participants in at least one week-long STEM summer camp. All families with children who were in our target age range and had signed up to participate in a STEM summer camp were sent information about the study. Those who expressed interest in having their child participate were sent links which directed them to the consent and assent forms, and questionnaire, and behavioral tasks, all of which were conducted online. We sent a $10 USD gift card to each participant as compensation for their time. We did not collect gender data for this sample.

### 10.2 Measures

The CT measures used in this task were the same ones used in Study 2 adapted for online data collection. The same scoring methods for the CT measures that were specified in study 2 were used in this study. In addition to the CT measures, participants answered questions about their previous programming experience in a short questionnaire. In it, participants were asked to “select all of the programming platforms, games, and languages” they had previously used from a list that included Scratch, Bitsbox, Hour of Code, Agent Cubes, Alice, Blockly, Code.org, Lightbot, Code Combat, Bloxels, Python, and Javascript. They were also permitted to write in other programming related activities that were not listed. The programming experience measure we use in the analysis of this experiment is calculated by summing the total number of different programming platforms participants reported using, including items in the list we provided and their write-in answers.

### 10.3 Procedure

After completing parental consent and child assent forms, children completed a short Qualtrics questionnaire about their experience with programming with the help of their parents. After completing the questionnaire, participants were directed to Pavlovia.org where the online CT measures were conducted.

## 11 Study 3 results

**Figure 8:**
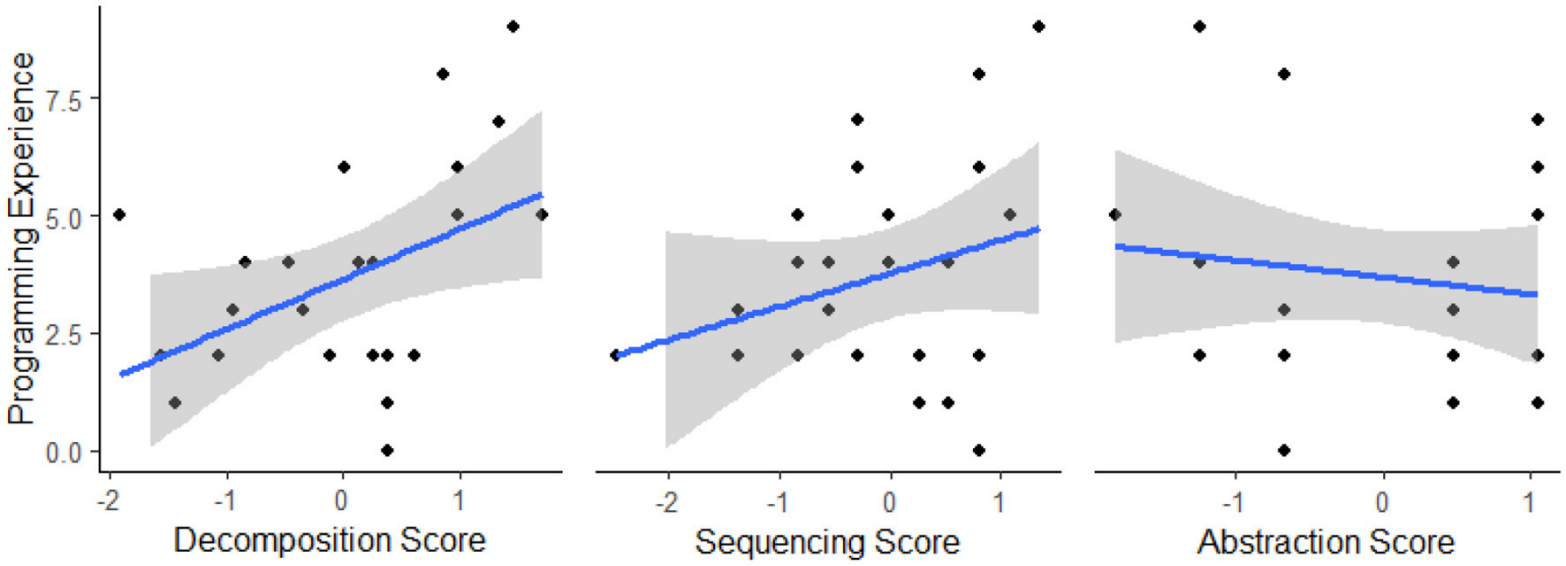
Scatterplots illustrating the relationship between programming experience and standardized CT measure scores in study 3. Gray shading represents the 95% confidence interval.

The aim of this experiment was to examine the relationship between programming experience and CT scores. Toward this end, we use a multiple regression analysis fitting programming experience to predictors representing main effects for each of the CT measures as well as a predictor for the main effect of age. The scores of each CT measure were standardized using *z*-score transformations and age was mean-centered. One participant was excluded from the analysis for missing data due to them skipping an item in the CT measures. While holding fixed the main effects of sequencing, abstraction, and age, the model reveals a significant main effect of decomposition β = 1.18, *t*(19) = 2.14, *p* = 0.045 (see Figure 8). No significant main effect of sequencing score (*p* = 0.240), abstraction score (*p* = 0.774), or age (*p* = 0.621) were revealed. Correlations between CT measures revealed no relationship between decomposition scores and sequencing scores *r*(23) = –0.07, *p* = 0.744 or between sequencing and abstraction scores *r*(23) = –0.07, *p* = 0.730. A moderate negative relationship was revealed between decomposition and abstraction scores *r*(23) = –0.41, *p* = 0.045.

## 12 Study 3 discussion

This final study reveals a similar pattern of results to the previous two studies. Using a separate experience-based measure of programming in place of an ability-based measure, we find that the measure of decomposition is once again the only one that reliably correlates. Together with the findings from the previous studies, we interpret this result as converging evidence that decomposition represents a prevalent cognitive component of CT development.

## 13 Implications and limitations

We set out to test the degree to which programming proficiency correlates with cognitive measures theorized to index three skills that are regularly used by the CS education community to describe CT: decomposition, sequencing, and abstraction. The present work reveals converging evidence over three studies that decomposition, as measured by the “Block Design” task from the WISC-III, correlates most reliably with both programming proficiency and programming experience out of the three CT components tested. One possible explanation for the fact that our measures of sequencing and abstraction are not found to significantly relate to programming proficiency is that they do not successfully capture the targeted cognitive components of CT. That is, the Picture Arrangement and Sequential Order measures may not effectively index sequencing skill and the Repeated Patterns task may not index abstraction as well as we theorized they might. This was a possibility from the outset, given that validity of these measures for capturing CT had not been established prior to this study. Alternative measures attempting to isolate the cognitive components of sequencing and abstraction might yield different results. However, an additional limitation has to do with the fact that the sources of our participants in these studies are families associated with community partners, namely a local summer camp and a class of fourth grade students at a local elementary school. Therefore, our sample size is limited first by the finite number of children who are involved in these programs, and second by the number of families who opt into our study out of those who are involved in these programs. These limitations result in small sample sizes and relatively low statistical power. For this reason, we are hesitant to draw strong conclusions about the lack of a relationship between any cognitive components and programming proficiency based on our results. Further, it is important to consider these findings within the context of developmental change.

Cognitive components of CT may not develop in a simultaneous or uniform manner as programming experience is gained (Tsarava et al., 2022). The present studies examine the relationship between CT components and programming proficiency in children ages 7–12. Although abstraction and sequencing were not found to correlate with programming proficiency in these studies, it is possible that they emerge at other stages of CT development. In the case of sequencing, for example, children of this age have already spent years in school learning sequencing skills through following instructions and story-telling activities. That is, the development of sequencing skills may reach a plateau by this stage, whereas children at earlier stages of development may still be growing in their ability to sequence, therefore being more likely to exhibit more variability in sequencing skills reflecting their individual experiences. In fact, Kazakoff et al. (2012) reports significant gains in sequencing ability as a result of robotics training among kindergarten students, a population much younger than the ones we draw from for this work. Conversely, the abstraction skill may be somewhat more developmentally advanced than the other two components measured here, such that children at this developmental stage lie at a relatively low plateau of abstraction ability that may precede a substantial improvement in years to come. Thus, the correlation revealed between decomposition and programming proficiency could indicate an intermediate level of CT development within our sample characterized by growth of decomposition skills and relatively static sequencing and abstraction skills. This description of subsequent stages of sequencing, decomposition, and abstraction development represents just one possible way that various stages might progress. CT development would also likely include many of the CT components that did not fall within the scope of this work. The possibility of this complex development pattern highlights the importance of measuring various cognitive components of CT individually and at different stages of development.

The Lightbot game, having been designed as a teaching tool rather than an assessment tool, also reflects the stages of CT development detailed above in its level design. Sequencing elements, for example, are present throughout the game from the first level, wherein the player will learn that they need to navigate their avatar to stand on a goal destination before using the command to light the goal and complete the level. Soon after, the game begins to place more emphasis on decomposition with more complex levels that encourage the player to break down the level space not only into individual spaces, but also sets of spaces, in order to progress efficiently. For example, level five includes two goal spaces, the second of which can be reached using a sequence identical to that used to reach the first. A child with a high level of decomposition ability will recognize that the problem can be broken into two smaller, identical problems and progress through this level more quickly than children with a lower degree of decomposition skill. At this point in the game, both sequencing and decomposition are needed, but if there were no problems progressing through the first few levels, then decomposition is more likely to be the limiting factor here. The abstraction elements of the game come into play last according to the Gouws et al. (2013) analysis, which concluded that abstraction is less prominently represented until the introduction of functions, which does not occur until the game's ninth level. Few of the participants were able to reach the ninth level within the relatively short time cutoff used for the programming proficiency measure. Even among those who were eventually able to reach it after the time cutoff, only one progressed past level nine by more than three additional levels. Thus, it seems that multi-stage development of CT-related cognitive abilities is reflected in the progression chosen by Lightbot game designers to teach crucial coding concepts to its players.

Further research examining the development of CT across an extended period of programming training is required to directly test this multi-stage theory of CT development. Initially, the second study presented here was intended to do just that by using a pre-post design to study programming and CT development throughout a semester of CS education. Unfortunately, the COVID-19 pandemic prevented the collection of post-test data. Although we were unable to establish a causal relationship, the finding that decomposition correlated with programming proficiency in all three studies carries meaningful implications for the assessment of CT, as well as the instruction of CS, on its own. The existence of a programming-independent index of CT would allow for its measurement whether the child in question has a substantial amount of programming experience or not. It would also be useful for comparing the CT ability of children with experience in different programming languages or comparing the contributions of specific CS education programs to the development of different components of CT. From an educational perspective, it may be beneficial for interventions aimed at improving CT in this age range to incorporate programming as a means to improve decomposition skills while other supplementary activity may be required to target other CT components.

It is worth noting that this work revealed a relationship between programming proficiency and a single measure of decomposition. It is an open question as to whether the this relationship would hold for other decomposition measures, specifically those that draw less heavily on spatial reasoning. Previous work has shown that certain general intelligence factors including spatial reasoning as well as fluid reasoning and working memory correlate with CT ability (Ambrósio et al., 2014; Román-González et al., 2017). As mentioned in Section 2, these cognitive constructs are so broad that it is difficult to say whether CT correlates with general intelligence as a whole or if it selectively relates to narrower subdivisions of it. Such broad constructs also inherently involve multiple CT components, making them poor measures of individual aspects of CT. Despite our attempt to adopt measures that more selectively target specific components of CT, the studies reported here suffer the same difficulties to some, though hopefully a somewhat lesser, degree. For example, although we selected the “Block Design” task for the fact that it requires decomposition-related problem-solving strategies, it also requires spatial reasoning ability. In fact, in the context of the WISC-III battery from which it was taken, the “Block Design” task was designed to measure visuo-spatial reasoning. Therefore, in the first two studies presented here, there is no means to separate the contributions of decomposition and spatial reasoning in the “Block Design"-Lightbot relationship. The results of the third study, however, show that performance on the “Block Design” task also correlates with an experience-based programming measure that is independent of participants' spatial reasoning abilities, suggesting that the results of the previous studies do not solely reflect the shared spatial reasoning elements of the two tasks.

## 14 Conclusions and further research

The present work builds on previous work testing the theoretical claims of CT transfer effects by examining how programming proficiency relates to specific cognitive components of CT. We have identified at least one cognitive component that, measured using a task from a cognitive testing battery, reliably coincides with both programming proficiency and quantity of programming experience. This work represents a first step toward investigating CT transfer by examining CT at the level at which it is described in the CSEd literature.

Future work should explore other cognitive measures as indexes of abstraction and sequencing, given the possibility that ours failed to capture those target cognitive CT skills. Other measures of decomposition and programming proficiency should also be tested. To extend on the work presented here, for example, a measure of decomposition that is less reliant on spatial reasoning could be used to tease apart the relationships between visuo-spatial ability, decomposition skill, and programming proficiency in this age range. In addition, for a clearer understanding of the nuances of CT development, future research should extend this work by examining a wider range of basic cognitive components and at various stages of CT development. To understand the impact of the ubiquity of CSEd in early school years, it is important to continue to improve our understanding of the development of the basic cognitive components that comprise Computational Thinking.

## Data Availability

The raw data supporting the conclusions of this article will be made available by the authors, without undue reservation.

## Footnotes

^1^ https://lightbot.com

^2^This study was designed to replicate the pre-post analysis of Study 1 in elementary school students during a semester of CSEd. However, the COVID-19 pandemic prevented collection of post-test data making pre-post analysis impossible.
